# Force Generation upon T Cell Receptor Engagement

**DOI:** 10.1371/journal.pone.0019680

**Published:** 2011-05-10

**Authors:** Julien Husson, Karine Chemin, Armelle Bohineust, Claire Hivroz, Nelly Henry

**Affiliations:** 1 Institut Curie, Centre de Recherche, Paris France; 2 CNRS, UMR168, Paris, France; 3 INSERM, U932, Paris, France; 4 Université Paris 6, Paris, France; Institute of Microbial Technology, India

## Abstract

T cells are major players of adaptive immune response in mammals. Recognition of an antigenic peptide in association with the major histocompatibility complex at the surface of an antigen presenting cell (APC) is a specific and sensitive process whose mechanism is not fully understood. The potential contribution of mechanical forces in the T cell activation process is increasingly debated, although these forces are scarcely defined and hold only limited experimental evidence. In this work, we have implemented a biomembrane force probe (BFP) setup and a model APC to explore the nature and the characteristics of the mechanical forces potentially generated upon engagement of the T cell receptor (TCR) and/or lymphocyte function-associated antigen-1 (LFA-1). We show that upon contact with a model APC coated with antibodies towards TCR-CD3, after a short latency, the T cell developed a timed sequence of pushing and pulling forces against its target. These processes were defined by their initial constant growth velocity and loading rate (force increase per unit of time). LFA-1 engagement together with TCR-CD3 reduced the growing speed during the pushing phase without triggering the same mechanical behavior when engaged alone. Intracellular Ca^2+^ concentration ([Ca^2+^]_i_) was monitored simultaneously to verify the cell commitment in the activation process. [Ca^2+^]_i_ increased a few tens of seconds after the beginning of the pushing phase although no strong correlation appeared between the two events. The pushing phase was driven by actin polymerization. Tuning the BFP mechanical properties, we could show that the loading rate during the pulling phase increased with the target stiffness. This indicated that a mechanosensing mechanism is implemented in the early steps of the activation process. We provide here the first quantified description of force generation sequence upon local bidimensional engagement of TCR-CD3 and discuss its potential role in a T cell mechanically-regulated activation process.

## Introduction

T cell activation is a crucial event in the development of adaptive immune response to pathogens or tumor cells. Activation is triggered as a T cell encounters an antigenic peptide associated with the major histocompatibility complex at the surface of an antigen presenting cell (APC). This encounter is followed by the formation of a dynamic contact zone called immunological synapse (IS). T cell activation triggering is a highly sensitive and specific process involving several pairs of ligands and receptors in addition to the central TCR-antigen engagement. Among these molecules, the integrin lymphocyte function-associated antigen-1 (LFA-1) plays a crucial role, as it controls T cell adhesion to APC and formation of long lasting contacts [1], [2].

While signaling pathways are now known with increasing details [3], the question of the mechanism of TCR triggering remains unclear despite its critical importance. A number of models have been proposed putting forward thermodynamical, kinetic, conformational or theoretical considerations, none of which integrating the whole set of available data [4]. Lately, the idea began to form that a link might be missing in the comprehensive vision of the process, due to almost complete oversight of its mechanical aspects. This hypothesis was first simply evoked as a possible working hypothesis to reconcile binding data with activation profiles [5], [6]. The involvement of mechanical forces in the triggering process was also put forward relatively early as a possible driving force in models assuming TCR-CD3 conformational changes [7]. More recently, Ma and coworkers proposed a detailed TCR deformation model where mechanical stress could induce conformational changes that would unmask sites of phosphorylation and allow TCR signaling [8]. Furthermore, two recent studies have proposed that TCR/CD3 itself acts as a mechanotransductor [9], [10] in response to external forces, thereby contributing to the paradigm of forces as integral part of TCR triggering. However, up to now the sources of mechanical forces have only been hypothesized, supposedly originating from membrane tension due to bidimensional alignment of proteins of different size in the cell-cell contact zone [11] or from cytoskeletal activity associated with T cell motility [8].

In order to gain insight into the nature of the forces potentially exerted by the T cell during antigen recognition and activation, we implemented a biomembrane force probe (BFP) technique first developed by Evans and coworkers [12] coupled with a simplified model APC. The latter consisted of a micrometric bead coated with antibodies against defined receptors of T cells. Primary T cells were chosen over model tumor T cell lines, as these may present particularities due to their tumorogenic nature. Micrometric beads were coated with antibodies specific for the TCR-CD3 complex and/or LFA-1 molecules. We have previously shown that this model consistently echoes the biological situation [13]. In addition, the engagement of these two critical surface receptors has been shown to reproduce the immune synapse pattern observed in entire cells systems [11], [14]. As the BFP technique is coupled to optical microscope, we have been able to image in real time both cell morphology and fluorescent labeling. Then, in parallel to force generation, we have followed Ca2+ signaling as an early signature of T cell commitment into the activation process, and actin polymerization to evaluate cytoskeleton involvement in the mechanical events. As BFP holds a mechanical transducer with tunable stiffness, we also examined the possible dependence of T cell mechanical behavior on the stiffness of the interacting object.

Our results provide evidence that local bidimensional engagement of TCR/CD3 specifically triggers intense T cell mechanical activity consisting in a sequence of pushing and pulling forces against the model APC. In order to better understand this force generation, we have quantified the different phases analyzing both pushing structure velocity and pulling force loading rates (force increase per unit of time) upon engagement of different surface receptors. We eventually discuss these results in the light of Ca^2+^ synchrony and actin polymerization events, and the potential role of force generation in T cell activation.

## Results

### T cell mechanical response to TCR-CD3 engagement

In order to substantiate the idea of a defined T cell mechanical behavior associated with activation triggering, we brought into contact a primary CD4+ lymphocyte with a simplified model of an APC by using an adapted version of the BFP. The model APC consisted of a 2.8 µm in diameter bead coated with antibodies against the CD3ε subunit of TCR/CD3 receptor. This enabled to engage surface receptors in well-defined molecular and mechanical conditions. In the meantime, we followed [Ca^2+^]_i_ to certify the actual commitment of the cell into the activation process.

**Figure 1:**
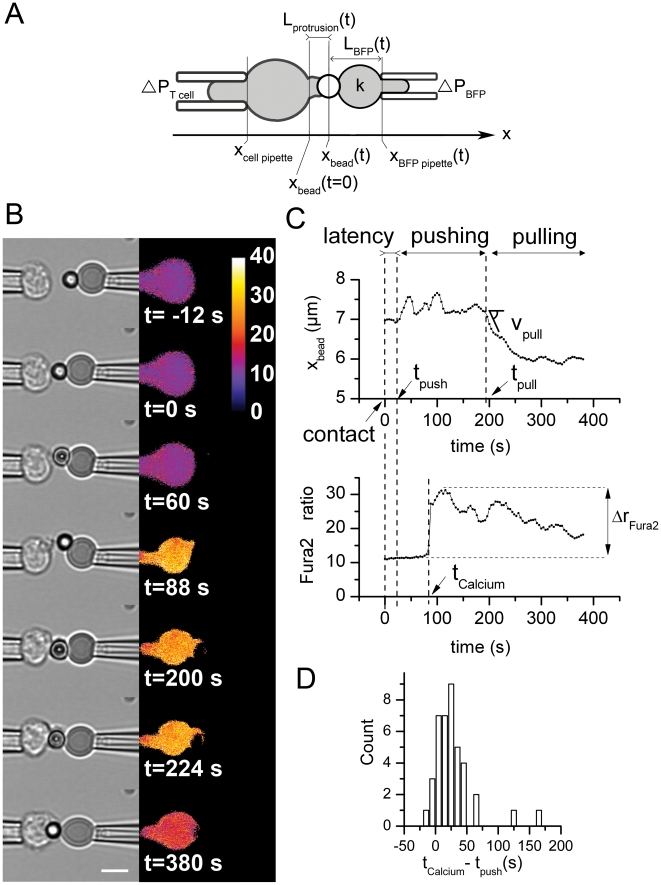
Successive phases during the interaction of a T cell with an anti-CD3 coated bead on the biomembrane force probe. (A) Schematic view of the experimental setup. A T cell (left) is held by a micropipette, which extremity is at a fixed position x_cell pipette_ along the x-axis, with an aspiration pressure ΔP_T cell_. The BFP (right) consists in a bead (centered at x_bead_(t)) adhering to a red blood cell, which is maintained by a micropipette with an aspiration pressure ΔP_BFP_. This aspiration sets the red blood cell stiffness k. The position of the right micropipette edge is located at x_BFP pipette_(t). After making contact with the bead at time t = 0, the T cell emits a protrusion of length L_protrusion_(t) = x_bead_(t)-x_bead_(t = 0). (B) Representative experiment (Movie S1) with a static BFP-holding micropipette (Left: brightfield images; Right: Fura2 ratio). Bar is 5 µm. (C) Above: Position x_bead_(t) during the three consecutive phases. During the “latency phase” the bead is immobile. At t = t_push_, the “pushing phase” starts as a protrusion emerges from the T cell, leading to x_bead_(t)>x_bead_(t = 0). The “pulling phase” starts at t = t_pull_, when the protrusion retracts and the T cell pulls on the bead. Below: Fura2 ratio versus time. The ratio increases abruptly at t = t_Calcium_, and t_Calcium_>t_push_. (D) Delay t_Calcium_−t_push_ between the onset of protrusion growth and Fura2 ratio increase.

In order to validate the potential of activation of the beads used in this study, we activated the CD4+ primary T cells with these beads and checked the phosphorylation of signaling molecules known to be involved in the TCR/CD3 signaling cascades. The anti-CD3 coated beads induced the tyrosine phosphorylation of the Src family of proteins, of the ZAP70 kinase, which binds to the CD3 complexes, of the LAT adaptor, which is one of the substrate of ZAP70 and of the MAPK ERK1 and ERK2 [3]. Uncoated beads did not induce any phosphorylation (Fig. S1A) demonstrating that the anti-CD3 beads were indeed able to induce activation of the TCR/CD3 signaling cascade in CD4+ T cells. Moreover, these beads were able to induce some functions in CD4+ primary T cells as shown by the induction of IL-2 secretion by the activated T cells (Fig. S1B). Altogether, these results show that the beads coupled to the BFP probe and used in this study induce functional activation of T cells. A T cell was held in place on the left-hand side at the end of a micropipette. The BFP, which was made by coupling a coated bead to a red blood cell (RBC), was held by an opposite pipette on the right-hand side (Fig. 1A). In this first set of experiments, the bead was brought into contact with the T cell by soft and uniaxial motion of the BFP-holding pipette, up to the overlap of the dark fringe of the bead with the cell rim. The formation of physical contact between the cell and the bead was determined by a minute compression of the probe corresponding to less than one-pixel (i.e. 100 nm) decrease of the BFP length on the microscope image which indicated that the contact occurred at a low force smaller than 5 pN. This stated the arrest of the right pipette translation and set the contact time or time zero of the experiment. In this static protocol, no further motion was applied on the pipettes by the operator and the cell response was monitored in this geometry. Time-lapse bright field and fluorescence images were acquired reporting cell morphological alterations, BFP dynamics and intracellular calcium concentration changes induced by T cell-artificial APC contact.

We found that the T cell response to the contact with the BFP-held anti-CD3-coated bead consisted in a sequence of three consecutive phases that we called the latency phase, the pushing phase, and the pulling phase, respectively (Fig. 1B, C).

The latency phase, which corresponded to a silent phase where nothing happened, lasted 45±7 s after cell-bead contact was attested (Table 1).

**Table 1:** Kinetics upon TCR-CD3 engagement.

	Latency phase	Pushing phase	[Ca2+]i increase	Pulling phase	Engulfment
Frequency	100%(N = 48)	98%(N = 48)	88%(N = 48)	73%(N = 48)	86%(N = 22)
starting time/duration	t_contact_ = 0 sduration 45+/−7 s	t_push_ = 45+/−7 sΔt_push_ = 140+/−15 s	t_Calcium_ = 88+/−20 s	t_pull_ = 182+/−15 s	within 500 s

t_contact_: contact time of the initial T cell-bead contact. t_contact_ is taken as time reference t = 0.

t_push_: beginning time of the pushing phase (relative to t_contact_ = 0). t_push_ also marks the end of the latency phase.

Δt_push_: duration of the pushing phase, Δt_push_ = t_pull_−t_push_.

t_Calcium_: starting time of the time of the [Ca2+]i increase.

t_pull_: beginning time of the pulling phase (relative to t_contact_ = 0).

The pushing phase consisted of the growth of a directional cell protrusion initially emerging between the cell body and the bead. The beginning of this phase (at a time t_push_ relative to the contact time t = 0) was reported by an initial axial compression of the RBC in response to the pushing force generated by the protrusion onset. The pushing force quickly led to BFP buckling and breakage of the axial-symmetry with protrusion extension out of BFP axis. The initial RBC axial compression was typically inferior to 0.5 µm, which for a probe stiffness of 50 pN/µm corresponded to forces around 25 pN. Average duration of this pushing phase (Δt_push_) was found equal to 137 s (Table 1).

[Ca^2+^]_i_ peaked during this pushing phase for most cells, as measured with the Fura-2 ratiometric calcium probe. The triggering of the transient calcium wave, witnessing T cell activation, occurred at a time t_Calcium_, which was larger than t_push_ as shown in Figure 1D. However, t_Calcium_ and t_push_ were weakly correlated (correlation coefficient ≈0.44).

In most cases, at a given time t_pull_ (see Table 1), the cell machinery appeared to reverse switching to the pulling phase characterized by protrusion retraction and generation of pulling forces as reported by the elongation of the BFP transducer ΔL_BFP_ (Fig. 1, Movie S1). This protrusion retraction ended with the engulfment of the bead by the T cell in the majority of the cases within the duration of the experiment. This typically lasted 500 s.

We checked using silica beads which form non-specific adhesive contact with the T cell, that neither mechanical activity nor [Ca^2+^]_i_ increase were elicited in the absence of specific receptor engagement at the cell surface (Fig. S2).

These results clearly demonstrate that engagement at cell surface of the TCR/CD3 complex triggered not only a complex biochemical cascade, but also an active mechanical response (schedule and statistics recapitulated in Table 1).

Next, to shed more light on this mechanical process we underwent the quantification of the forces developed by T cell in pushing- and pulling- phase upon TCR-CD3 surface engagement.

### Pushing phase upon TCR-CD3 engagement

Pushing phase started with a short episode of BFP compression before the probe quickly bent off-axis for pushing forces of the order of 25 pN (0.5 µm compression length, 50 pN/µm probe stiffness). Higher compression forces have been measured by Heinrich and coworkers [15], [16] using a RBC pushed against a planar wall [13]. There are possibly two reasons for the quick bent off-axis observed here for low pushing forces. First, this might be due to the different geometry implemented in our experiment. Indeed, compression takes place not on an infinite plane but on the tip of the micron-size bead attached to the apex of the RBC, which could induce an early elastic instability. Second, the protrusion emerging from the T cell did not necessarily pushed along the probe axis (x-axis), which could also generate out-off axis RBC deformations.

**Figure 2:**
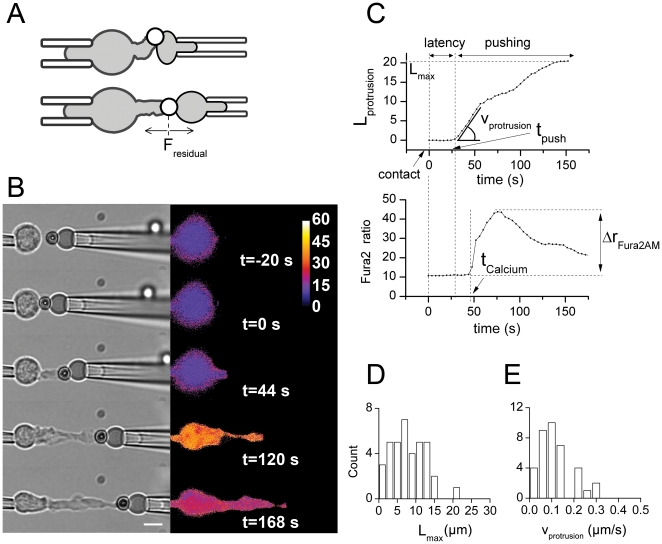
Characterization of the CD3-induced pushing phase. (A) The dynamic probe-protocol used to measure T cell protrusion growth consists in stepping back the right pipette along the x-axis as soon as a RBC compression is detected (above), in order to relax this compression (below). The residual force exerted on the T cell protrusion after stepping back the pipette, F_residual_ , is kept below 25 pN. (B) Representative experiment (Movie S3) using the dynamic-probe protocol (Left: brightfield images; Right: fura2 ratio). Bar is 5 µm. (C) Above: Protrusion length L_protrusion_ versus time. During the latency phase, L_protrusion_ is constant and equal to 0. The pushing phase starts at t = t_push_, when L_protrusion_ starts increasing with time. L_protrusion_ increases initially at a constant velocity v_protrusion_. The protrusion reached a maximal length L_max_. Below: fura2 ratio versus time. The ratio increases abruptly at t = t_Calcium_>t_push_, with a maximal amplitude Δr_fura2_ relative to the base level. (D) Distribution of L_max_ over N = 37 cells. L_max_ = 8.2+/−0.7 µm. (E) Distribution of v_protrusion_ over N = 37 cells. v_protrusion_ = 0.12+/−0.01 µm/s.

The probe bending (Fig. 1B, and for a more dramatic off-axis deformation, see Movie S2) prevented further measurement of the pushing force. In order to still carry on pushing phase quantification, we decided to use an alternative strategy. It consisted of progressively stepping back the micropipette holding the BFP as the cell protrusion grew. This provided a mean to keep uniaxial on-focus protrusion growth, thus enabling protrusion length (L_protrusion_) measurement. This so-called dynamic-probe protocol consisted in a two-step cycle aiming to maintain BFP transducer around zero-force, exerting no force on the bead, then no force on the cell protrusion according to principle of action and reaction (Fig. 2A–B, Movie S3). The anti-CD3 BFP-held bead was first brought into contact with the T cell as described just above. Then, as soon as RBC compression was detected, the right pipette was stepped back along the *x*-axis by the operator up to the detection of the minimal RBC elongation. Minimal detection threshold corresponded to one-pixel displacement of bead, i.e. compression or tensile forces lower than 10 pN for a 50 pN/µm probe stiffness. This dynamic-probe-protocol was performed in real-time by the operator through eye-detection and controlled *a posteriori* using the bead tracking procedure described in the methods section, which confirmed that the residual forces applied on the probe in the two-steps-cycle step back of the pipette were kept below 25 pN. We further checked that fluctuations of the residual force reaching up to 75 pN for a short period of time did not alter the average growth speed of the protrusion (Text S1, Fig. S3, and Movie S4).

Thus, we measured the protrusion length emitted by the T cell as a function of time for a series of 37 cells. Initial growth was characterized by a constant growth speed. We measured this linear growth for each cell by fitting a linear segment of the first 50 s of the protrusion growth (Fig. 2C). After this time, some cells immediately arrested, some stalled during several tens of seconds before arrest and some went on with constant growth velocity up to arrest. This variability was reflected by protrusion maximal lengths (L_max_) distribution shown in Figure 2D. The initial growth velocity (v_protrusion_) distribution is shown in Fig. 2E. The average values were found equal to and L_max_ = 8.2+/−0.7 µm and v_protrusion_ = 0.12+/−0.01 µm/s.

The T cell was then able to push the model target forward with an initial constant velocity during a few tens of seconds.

### Characterization of the CD3-induced Pulling Forces

**Figure 3:**
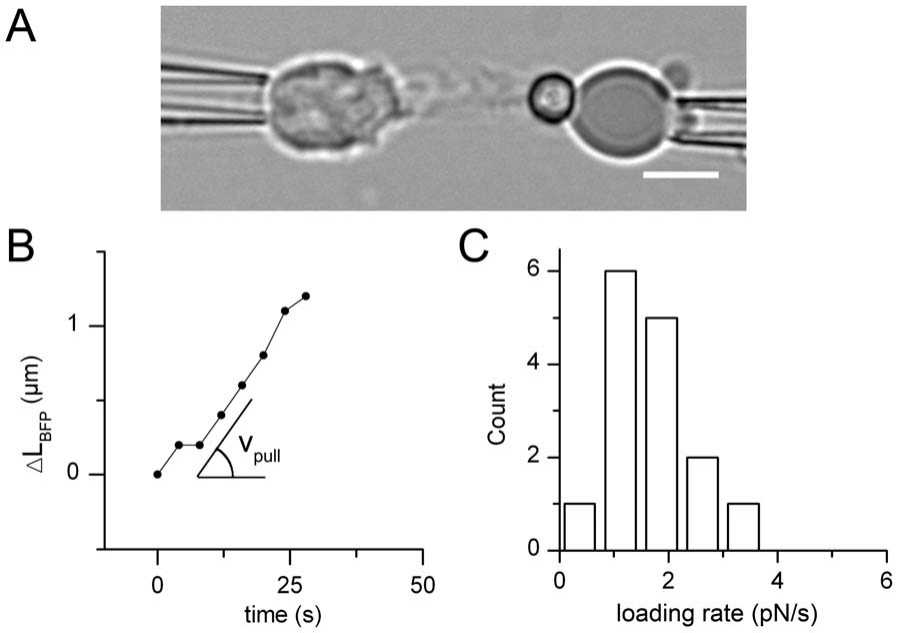
Characterization of the CD3-induced pulling phase. (A) During the pulling phase, the T cell protrusion pulls on the bead of the force probe, leading to its elongation over time. Bar is 5 µm. (B) elongation of the BFP ΔL_BFP_(t) = L_BFP_ (t)−L_BFP_ (0) in a representative experiment. ΔL_BFP_ increases initially at a constant speed v_pull_ = dL_BFP_ (t)/dt. (C) Distribution of the resulting loading rate r = dF/dt (expressed in pN/s), r = 1.6+/−0.2 pN/s for a probe stiffness k = 50 µm/s (N = 15 cells).

The third phase (the pulling phase) started as cell protrusion forces exerted on the model bead switched from pushing forces — transduced by force probe compression — to pulling forces reported by force probe elongation (ΔL_BFP_). In this phase, the BFP-holding micropipette was kept static as soon as the bead started being pulled toward the T cell body until the end of the experiment. In order to determine the initial pulling force loading rate, r = dF/dt, developed by the T cell against the target bead, the force transducer elongation was measured as a function of time in the initial stage of this pulling phase for a given probe stiffness equal to 50 pN/µm. Measurements were performed within the linear regime of the force-elongation relation, i.e. for elongations smaller than 1 µm as shown by the two-probes test (see materials and methods section). The results indicated that a constant pulling rate, i.e. a pulling force increasing linearly with time, was set up by the cell within the first thirty seconds of this process (Fig. 3, Movie S5). A linear regression of the elongation versus time data was collected on 15 cells (Fig. 3C) and provided a mean loading rate value equal to 1.6+/−0.2 pN/s. No further measurements were collected after the first 30 seconds since the probe elongation above 1 µm deviated from linear regime. After a longer period of time, the cell went on pulling, often dragging the probe out of the pipette and eventually partially engulfed the target particle.

This demonstrated that TCR-CD3 engagement at the surface of T cell by a model target cell triggered constant pulling loading rates of the order of 2 pN/s.

### Reduced CD3-Dependent Force Generation upon CD18 Engagement

#### LFA-1 alone

**Figure 4:**
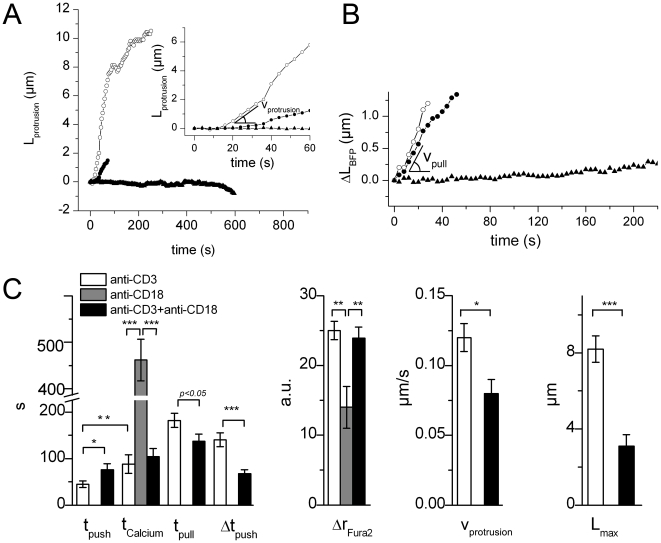
Reduced CD3-Dependent Force Generation upon CD18 Engagement. (A) L_protrusion_ versus time for anti-CD3 coated beads (open circles), anti-CD3+anti-CD18 coated beads (full circles), and anti-CD18 coated beads (full triangles). The co-engagement of both CD3 and CD18 reduces v_protrusion_, while CD18-only engagement abolishes protrusion growth. Inset shows the beginning of the growth. (B) BFP elongation ΔL_BFP_ versus time, for anti-CD3 coated beads (open circles), anti-CD3+anti-CD18 coated beads (full circles), and anti-CD18 coated beads (full triangles). (C) Comparison of outcome upon CD3-, CD3+CD18- and CD18-engagement when T cells interact with the BFP. We compare times (t = 0 when the bead contacts the T cell) t_push_ (start of the pushing phase), t_Calcium_ ([Ca^2+^]_i_ increase time), t_pull_ (start of pulling phase), and duration of the pushing phase Δt_push_ = t_pull_−t_push_. We also compare the Calcium response amplitude (fura2 ratio Δr_Fura2_), pushing speed v_pull_ and maximal protrusion length L_max_ (^*^p<0.01, ^**^p<0.001, ^***^p<0.0001).

Integrin engagement has been shown to play an important role in T cell/APC activation and immunological synapse formation as well as in force generation triggering [1], [2], [14], [17]. We thus studied if integrin engagement by model APC at the surface of the T cell led to force generation. To this purpose the BFP-held target bead was this time coated with LFA-1 ligands (anti-CD18 antibodies to the β_2_ chain of LFA-1) and brought into contact with the T cell just as described above with TCR-CD3 ligands. By contrast with this last case, LFA-1 engagement did not generate any pushing forces on the target bead. Only very low pulling loading rates were detected. This low-pulling phase extended weakly over several hundreds of seconds after contact. We estimated that the corresponding small force probe elongation could be considered as linearly increasing over the first 400 seconds, reporting a loading rate of the order of 0.2 pN/s, using a probe of stiffness equal to 50 pN/µm. The measured loading rate stayed low at other stiffnesses, but the detection limit of the experiment did not allow us to ascertain whether and how those low loading rates depended on the stiffness. This restricted pulling phase did not lead to any bead engulfment. Intracellular calcium concentration raised in 56% of the cases (10 cells out of 18) although with a lower amplitude than when the cells response was triggered by an anti-CD3 coated bead. Moreover, this calcium signal occurred at later time points than when T cells were activated through TCR-CD3 engagement — mean t_Calcium_ = 462+/−45 s for LFA-1 versus 88±20 s for TCR-CD3 engagement (Fig. 4C).

Hence, engagement of LFA-1 alone triggered neither pushing force nor engulfment of the target bead, although small pulling activity and restricted calcium signaling were detected.

#### Combined engagement of TCR-CD3 and LFA-1

We then studied if co-engagement of LFA-1 and TCR-CD3 at the surface of CD4+ T cells could lead to a different force-generation scenario. We thus coated target particles with both receptor ligands. We used the same surface density as for TCR-CD3 alone, and saturated the remaining binding sites with anti-CD18, which corresponded to a 6∶4 anti-CD3∶anti-CD18 ratio.

The three phases found upon engagement of TCR-CD3 alone were found again with the TCR-CD3/LFA-1 combination. However, the co-engagement induced a clear decrease of the protrusion length emitted in the pushing phase; averaged maximal length obtained according to the dynamic-probe protocol dropped to L_max_ = 3.1±0.6 µm, i.e. less than half value obtained upon engagement of TCR-CD3 alone (L_max_ = 8.2±0.7 µm). This was associated with a concomitant decrease of the growth velocity, which mean value was found equal to 0.07±0.01 µm/s while v_protrusion_ = 0.12±0.01 µm/s for TCR-CD3 engagement alone. Moreover, the pulling phase started earlier, and a fraction of the cells (5 out of 29, i.e. 17%) directly entered the pulling phase. The pushing phase duration, Δt_push_ = t_pull_−t_push_, dropped from 140±15 s for CD3 engagement alone down to 67+/−8 s (p<0.0001 using a two-tailed Student t-test, Fig. 4C). In addition, the protrusion morphology changed from a mainly “tube-like” structure to a shorter “cup-like” structure.

Calcium responses elicited upon combined engagement of TCR/CD3 and LFA-1 were unchanged. Both amplitude and triggering time-delay were similar to those induced by engagement of TCR/CD3 alone (Fig. 4C, Fig. S4).

The pulling phase developed with a probe stiffness of 50 pN/µm displayed the same characteristics than the ones observed engaging TCR-CD3 alone (Fig. 4B).

As for engagement of TCR-CD3 alone, the pushing-pulling sequence ended in target bead engulfment by the T cell.

The co-engagement of LFA-1 and TCR-CD3 receptors at the surface of the T cell reduced the pushing phase triggered upon the engagement of TCR-CD3 alone. However, the calcium signal and the pulling phase did not change.

### Target stiffness-sensitive pulling forces

**Figure 5:**
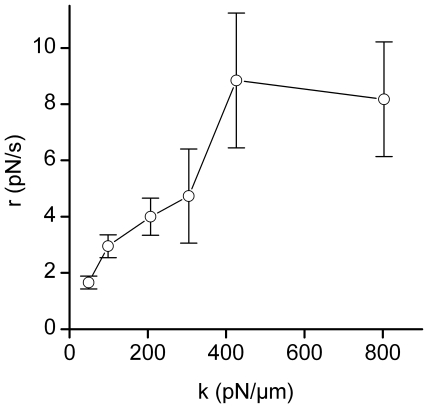
Loading rates generated by T cells depend on the Probe Stiffness. Loading rate r = dF/dt versus stiffness k of the force probe, for anti-CD3 coated beads.

Several cell types have been shown to adapt their mechanical or biological response to the stiffness of their environment [18], [19]. Thus, in order to investigate whether pulling forces exerted by T cells on the model APC depended on target stiffness, we varied the rigidity of the target-holding force probe from 50 to 1000 pN/µm using different aspiration pressures of the RBC. The induced probe elongation was measured as previously explained as a function of time to determine the initial loading rate, *r*. The variation of *r* as a function of probe stiffness is shown in Fig. 5 for TCR-CD3 engagement. The pulling loading rate increased with probe stiffness.

This suggests that T cells can adapt the forces generated by TCR/CD3 triggering to the mechanical properties of the APC.

### Molecular support to force generation

In order to check whether the force generation we observed was supported by actin polymerization, as shown for other mechanical processes [20], we performed the same experiments with cells previously treated for 20 min with 0.5 µM Latrunculin A. This inhibited actin polymerization. In this case, the pushing phase was totally abolished while very low pulling velocities (v_pull_ = 0.0012±0.0005 µm/s, N = 7) — more than one order of magnitude lower than the one obtained with untreated cells — persisted. The calcium signature was not significantly affected by Latrunculin A treatment (data not shown).

**Figure 6:**
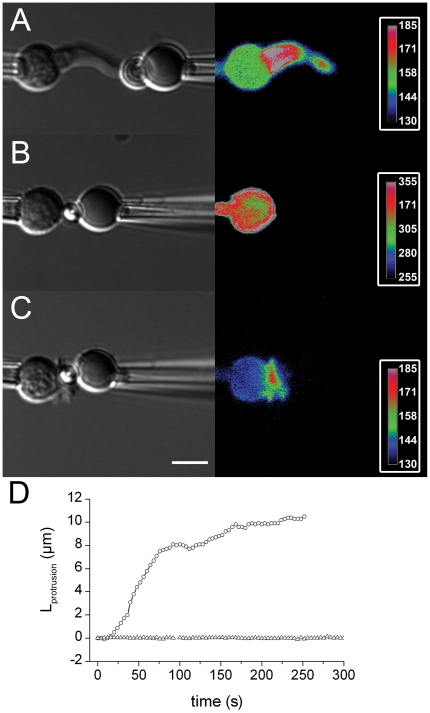
Cytoskeleton remodeling in T cells interacting with Abs-coated beads on the force probe. T cells were transfected with LifeAct-mCherry (left : DIC, right : color code mCherry). (A) Anti-CD3 coated bead. (see Movie S6. (B) Anti-CD18 coated bead. (C) Anti-CD3+anti-CD18 coated bead. (D) L_protrusion_ versus time for one representative experiment with a latrunculin A treated T cell (open triangles) or a non-treated T cell (open circles).

These results clearly indicated that actin polymerisation was required at least for entering the first phase, i.e. the establishment of the pushing phase. As well no significant pulling phase was observed possibly due to the absence of pushing phase. Doing the force generation experiment with LifeAct-mCherry transfected cells enabled to monitor the dynamic F-actin polymerization in the protrusion. The images shown in Figure 6 reveal the presence of a dense F-actin tube in the cell protrusion formed during the pushing phase when engaging TCR/CD3 (Movie S6, Movie S7, Movie S8).

In order to investigate the role of microtubule dynamics in the observed LT mechanical response, we measured the response of cells treated with inhibitory concentrations of Nocodazole and Colchicine. We observed the same qualitative response, i.e. a sequence of latency- pushing- and pulling- phase, ending with the anti-CD3 grafted microbead engulfment.

In order to confirm that protrusions were also observed without any micropipette involvement, we performed cell-particle contact in suspension and imaged the cell-particle conjugates after cell fixation (Text S1). We observed T cells presenting protrusions (Fig S5, top and middle), and T cells having internalized at least one bead (Fig. S5, below). Live observations of T cells in suspension in presence of anti-CD3 coated beads also demonstrated the presence of dynamic tube-like protrusions (Movie S9).

## Discussion

An increasing number of models propose that biomechanical processes underlay T cell activation. Yet, evidence is sparse. Thus, we developed an adapted BFP model using primary T cells and an artificial model APC coated with antibodies specific for the TCR/CD3 complexes and/or LFA-1 molecules. Thereby, we could achieve a local bidimensional surface receptors engagement in well-defined molecular and mechanical conditions and carry out real time imaging of the activation processes through visualization of [Ca^2+^]_i_ and actin polymerization.

We demonstrate here that upon engagement of TCR alone, T cells generate a reproducible sequence of pushing and pulling forces displaying characteristic cell protrusion growth velocity, force loading rate and duration. These force generation events occur after a short lag time independent of any prior engagement of adhesion receptors. The pushing phase onset even precedes intracellular calcium transient increase. Expectedly, actin polymerization supports the initial pushing phase as shown by Latrunculin A inhibitory effect. Engagement of LFA-1 molecules alone does not trigger such a sequence but modulates the TCR-CD3 pushing velocity.

The latency-pushing-pulling three-phases observed with our model setup closely reflects dynamical behavior observed in previous studies performed with entire cells. For instance, Negulescu and co-workers [21], described a three-stage contact between T cells and Ag-presenting B cells *in vitro*. A first contact phase, which consists of probing and contact with B cells prior to [Ca^2+^]_i_ increase (latency phase). This is followed by a recognition dynamic phase, which is characterized by extension of the T cell membrane toward the B cell and [Ca^2+^]_i_ increase (pushing phase). A third stabilization phase then follows, where [Ca^2+^]_i_ decreases and the T cell partially retracts on the B cell (pulling phase). The three-phase scenario also displays common features with phagocytic processes exhibited by neutrophils. Indeed, a succession of latency, membrane extension phase, where the cell body spreads over the bead [22] or the pathogen [23] and finally a third phase during which the neutrophil becomes round and the target is pulled inward. The three-phase dynamical behavior evidenced here is similar to the sequential T cell spreading reported by Bunnell and co-workers when Jurkat T cells were plated on anti-CD3 coated coverslips [24].

Looking in detail to the pushing phase, we have measured a cell protrusion growth initial velocity at zero-force load equal to 0.12 µm/s. This constant velocity is very close to the values reported for T cells crawling in vivo [25]. Interestingly, this is also similar to the velocity of a neutrophil membrane spreading over a yeast prior to engulfment [23], or to lamella advances in keratocytes locomotion [26]. In addition, polymerization kinetics calculated on the basis of the fundamental properties of dense actin filaments [27], [28] are also comparable. Hence, it seems that one of the early events triggered by TCR-CD3 engagement is the active directional polymerization of a dense actin network, i.e. a powerful force-generating system. The exact nature of the actin-membrane interaction enabling the protrusion to grow against the model-APC is not known. Yet, it is tempting to postulate that actin-TCR-CD3 physical coupling occurs via several molecular adaptors such as Ezrin/radixin/moesin proteins [29], [30] or formins [30]. This would create a (APC-ligands/TCR-CD3/actin network) physical link quickly put under tension by the forces generated by actin polymerization triggered upon TCR-CD3 engagement. Under this tensile force, any of the elements of the molecular assembling might respond by unfolding part of their structure to reveal a cryptic binding site or alternatively dissociate and break the link. This is similar to the TCR deformation hypothesis proposed by Ma and coworkers [8]. However, in their model, TCR binding *per se* does not trigger T cell activation, but provides the physical anchorage against which forces originating from the cytoskeleton can apply. These lead either to detach the contact in the case of too weak a binding, or to exert conformational changes of the TCR/CD3 complexes. These changes are seen, there, as the actual activation triggering event. We show here that TCR-CD3 engagement triggers a specific sequence of forces, which does not require prior cytoskeleton mobilization. The direct physical clamp of TCR-CD3 with both APC ligands and cytoskeleton opens multiple possibilities for regulatory processes and feedback loops. These depend on the properties of APC-T cell contact (number and density of engaged links, binding affinity, stiffness of the APC –see below) and on the actin-gel dynamics. The growth dynamics of this type of gel depend on the application of external forces as shown by in vitro experiments performed on biomimetic systems [31], [32]. Computational analysis of an actin protrusion growth by Atilgan et al. has shown that the speed of the protrusion depends directly on the number and spatial arrangement of actin bundles, but also how these bundles are tethered at the membrane. This may also underlie our observation that combined engagement of LFA-1 with the TCR-CD3 complex reduces the protrusion growth velocity. When activated integrins are engaged with the TCR, talin and other cortical actin binding proteins are recruited at the contact zone [33]. New links could then be created between actin and the plasma membrane.

Our results show that calcium response always followed the pushing phase, but the data did not really allow us to establish the synchronization of the intracellular signaling with force generation. The correlation coefficient between t_push_ and t_calcium_ had an intermediate value of 0.44, which does not provide a sharp conclusion with respect to the dependence of t_calcium_ on t_push_. This leaves open the hypothesis that independent clocks both synchronize on the contact time, with calcium triggering having a longer characteristic time. Moreover, [Ca^2+^]_i_ increases were observed in T cells treated by latrunculin A, which suppressed the pushing phase.

At a given point, protrusion growth stops and T cell starts to pull on the model APC with an initial constant loading rate. What controls this inversion of force generation remains unknown. Protrusion length, growth time or a given cell signal from the activation pathway could serve as an inversion signal. At that stage, whether the pulling phase has its own trigger or whether it is the continuation of the pushing phase is not known. In this respect, it should be noted that no significant pulling phase was observed in the absence of pushing. This pulling phase might constitute an additional level for the cell to evaluate the “quality” of the contact formed. It is also now accepted [34], [35] that force load modulates molecular interactions, in particular through exponentially increasing dissociation constants.

Alternatively, the pulling forces that we observed may drive intracellular exchange of membrane patches (also known as trogocytosis) that happens between T cells and APCs. It depends on actin polymerization [36], [37] and has been suggested to play a role in immune regulation.

We show that pulling forces exerted by the T cell upon TCR-CD3 engagement adapt to their environment. The loading rate during the pulling phase actually increased with probe stiffness. Many cell types have been shown to be sensitive to the stiffness and geometry of their environment from cell spreading [38], [39], cell polarity and migration [40], [41] to cell contractility [42], [43] and even cell differentiation [44]. Yet, to the best of our knowledge, this link between environment mechanical properties and cell behavior is established here for the first time on T cells. It remains that the mechanism by which cells are sensing stiffness is still far from being understood. Recently, Mittrossilis et al. [45] have demonstrated in myogenic cells a fast (<0.1 s) response to substrate stiffness changes. The speed of the response led the authors to conclude that the adaptation was mechanical in nature since implementation of a biochemical cascade would take longer. However, biochemical processes could also be very quickly induced, for instance by mechanosensitive channels opening that will directly impact the force generation process. Moreover, different cells may exhibit different adaptation mechanisms and timing. This TCR driven T cell adaptability to target stiffness may be required for the T cells to gain information from the interacting APC, in addition to biochemical signal transduction. Indeed, T cells are known to form different contacts and immunological synapses with different types of APCs, (reviewed in [46], which present different morphologies, probably different stiffnesses and induce distinct T cell functional responses. By recognizing its ligand at the surface of the APC, the TCR/CD3 complex might integrate several pieces of information, i.e. the presence of a specific antigen at the surface of the APC and the stiffness of the APC thus allowing an adapted immune response. Further investigation is needed to uncover the mechanosensing mechanism implemented in the T cell activation process. At this point, it is too early to see which mechanism is involved: a T cell activation-specific mechanism or an evolutionary universal mechanism present in all eukaryotic cells – including immunological cells – to evaluate the properties of their environment. There are similarities between the response to stiffness observed here and in myogenic cells by Mitrossilis and collaborators [43], suggesting that the latter mechanism is put into action.

The BFP technique, used in this study, allowed us to show that T cells can adapt the forces they exert according to the rigidity of the target they interact with. Yet, this setting did not permit to measure the effects of the target rigidity on later T cell activation events such as transcriptional activities. We will thus address this important issue by designing new experimental models.

In conclusion, the present study provides experimental evidence that in human primary T cells engagement of TCR/CD3 complexes initiates the generation of actin-dependent pushing and pulling forces that can adapt to the stiffness of the APC. These biomechanical processes, taking place at the very beginning of TCR-CD3 engagement, might support sophisticated controls of the exquisite sensitivity and specificity of the TCR for its ligand. The challenge is now to analyze in depth the molecular levers and regulations of this refine biomechanics.

## Materials and Methods

### Primary CD4+ T cells purification and transfection

CD4^+^ T cells were generated as in Miro and co-workers [47]. Briefly, CD4+ T cells were negatively selected from PBMCs, after depletion of CD14^+^ cells, using the T cell isolation kit II from Miltenyi Biotec. Sorted CD4^+^ T cells were 97–99% CD4^+^/CD3^+^. *Primary CD4+ T cells transfection*. Human fresh PBMC were transfected with 3 µg of a plasmid encoding the LifeAct-mCherry chimeric molecule, kindly given by Roland Wedlich-Soldner (Max-Planck Institute, Martinsried, Germany) using Amaxa Nucleofector technology (Köln, Germany) as described in [48]. Eighteen hours after transfection CD4+ T cells were purified by depletion with the isolation kit II from Miltenyi Biotec.

### Cytokine detection

Cytokine secretion was measured in the supernatants of 10^5^ CD4+T cells co-cultured for 18 h with 10^5^ beads coated as described in “Bead preparation” by ELISA using matched paired antibodies specific for IL-2 (DuoSet, R&D Systems, Minneapolis, MN).

### Cell extracts and immunoblotting

CD4^+^ T cells from (1.10^6^ cells/ml) were incubated for 15 min. at 37°C in RPMI. Activation was induced by adding 10^6^ beads at 37°C. Post nuclear lysates were prepared as in [48] analyzed under reducing conditions by SDS-PAGE and electroblotted on Immobilon P membrane (Millipore, Bedford). The following antibodies, anti-phospho-LAT (Y191), anti-phospho-p44/42 MAPK (Thr202/Tyr204), anti-PLCγ1, anti-phospho-Src family (Y416), anti-phospho-ZAP70 (Tyr319) were from Cell Signaling Technology. Anti-α-tubulin from Calbiochem. Secondary antibodies coupled to HRP were from Jackson Immunoresearch. The antibody/antigen complexes were visualized by an enhanced chemiluminescence detection system according to the manufacturer's instructions (ECL, Amersham-Pharmacia).

### Buffers

Phosphate buffered saline (PBS, pH 7.4, Invitrogen, France) and MS buffer (NaCl 140 mM, KCl 5 mM, MgCl_2_ 1 mM, CaCl_2_ 1 mM, Hepes 10 mM, BSA 0.5%) were used.

### Bead preparation

Streptavidin beads (Dynal, Invitrogen, France) 2.8 µm in diameter were coupled with biotinylated monoclonal antibodies (mAb) directed towards TCR/CD3 (mouse anti-CD3, UCHT1, Clinisciences, France) or LFA-1 (anti-CD18 antibodies to the β_2_ chain of LFA-1, Clinisciences, France). Coupling was performed using a 30 minutes incubation at room temperature in PBS. The beads were then washed with PBS and could be stored without altering the surface coupling over several weeks at 4°C. Antibody surface density was controlled using a fluorescent goat-anti-Mouse (GAM) antibody (FITC/Alexa 488-conjugated GAM, Invitrogen, France) and flow cytometry titration as described in Carpentier et al. [13]. Anti-CD3 and anti-LFA-1 beads exhibited mAb densities equal to 1.2±0.2 10^4^ mAb/µm^2^ and 2.0±0.2 10^4^ mAb/µm^2^, respectively. We also prepared hybrid beads bearing both ligands with the same anti-CD3 density, completed with anti-CD18 Abs, i.e. a 6∶4 anti-CD3∶anti-CD18 ratio. In this case, beads were first coated with anti-CD3, washed, and completed with anti-CD18.

### Chamber Preparation

Custom-built chamber consisted of a polydimethylsulfowxyde (PDMS)-walled (1 cm×2 cm) well ∼3 mm-thick, stuck to a 24×60 mm #1 glass coverslip with vacuum grease. Side walls were shaped with a tilt so that micropipettes could be introduced into the experimental chamber with a minimal angle (∼10 degrees). The experimental chamber was usually filled with 300 µL sample topped by ∼400 µL cell culture-compatible mineral oil in order to avoid evaporation during experiment. MS buffer was first introduced in the dry chamber. Next, micropipettes were filled with the same buffer and introduced into the chamber for several minutes before any experiment in order to allow BSA adsorption onto the glass surface, preventing adhesion with lymphocytes or red blood cells (RBCs).

### Micropipettes

Micropipettes were made from 1 mm (OD) glass-tube capillaries (Drummond scientific, US) pulled in a pipette puller (Sutter Instrument, US) to tip diameter in the range of 2 to 3 µm (ID). Three-axis microscrews (Newport, US) allowed T cell-holding pipette to be positioned, while a motorized three-axes micromanipulator (Sutter MP285, Sutter Instrument, US) was used to position and micromanipulate the BFP probe-holding pipette. The pipettes were connected to water reservoirs. The water level adjustment enabled to control aspiration pressure in the pipettes. Precision on this level was estimated to be on the order of 0.05 cm H_2_0, i.e. ∼0.5 Pa.

### BFP assembling

Before each T cell was used, a new BFP probe was formed. It consisted of a micropipette-held biotinylated RBC with a streptavidin-coated bead to it. These were prepared following the protocol used by Evans and co-workers [12]. Briefly, typically 2 µL of biotinylated RBCs and 2 µL of biotinylated Ab-coated streptavidin beads were injected in the experimental chamber. Next, an isolated bead was picked with the left pipette. A RBC was aspirated in the right pipette once desired suction pressure was set. Then, the bead was gently pressed at the apex of the RBC, after which the left pipette was retracted, leaving a BFP probe formed and ready to be used. Biotinylated RBCs were kindly provided by C. Gourier (Laboratoire de Physique Statistique, Ecole Normale Supérieure, Paris, France).

### BFP stiffness control

In this setup, the RBC serves as a spring of known stiffness, *k*, which can be tuned by the controlled aspiration pressure inside the micropipette holding the RBC according to the relation from references [49], [50]:
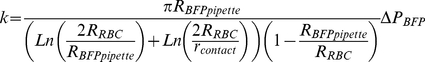
(1)where R_BFPpipette_ is the micropipette radius, R_RBC_ the radius of the part of the RBC remaining out of the micropipette, r_contact_ is the radius of the bead-RBC contact area, and ΔP_BFP_ is the aspiration pressure in the pipette. The geometrical parameters of the BFP, namely the pipette inner radius R_BFPpipette_, R_RBC_, and r_contact_, were measured on microscope images with a one-pixel, i.e. a 100 nm-precision using ImageJ software appropriate tool.

### BFP linear regime limit

In order to investigate the range over which the BFP behaved as a linear spring under elongation, i.e. that its stiffness k was independent of the RBC elongation, we introduced the following two-probe method. We stuck two RBCs diametrically opposite on a single streptavidin-coated bead (Fig. S4A). Each probe was held in a micropipette and aspiration pressures were set to obtain a stiff probe on the left side (k^stiff^ = 778 pN/µm), and a softer probe (k^soft^ ranging from 105 up to 422 pN/µm) on the right side. Tensile force was applied by retracting uniaxially the pipette and the elongation of each probe was measured. Plotting ΔL^soft^_BFP_ versus ΔL^stiff^_BFP_ enabled to establish that the linear regime persisted for elongations up to ≈1 µm (see Text S1 and Figure S7 for details).

### Tracking procedure

Protrusion lengths and RBC elongations were determined by tracking the position of the model bead with respect to the position of the fixed left micropipette on time lapse stacks of images. The tracking routine — implemented as a custom-programmed ImageJ macro [51] — was based on the fact that the bead exhibited on bright field microscope images an intensity profile showing a bright center and a dark edge at the radius of the particle. The tracking procedure consisted in determining the center of the bead by adjusting, on each image of the stack, the inverted-intensity of the image along four perpendicular radii of the bead to a Gaussian distribution in order to find the maximum intensity location along both x and y axes (Fig. S6). Running this procedure, the bead position was determined with a subpixel 30 nm-precision corresponding to forces of 1.5 pN precision for probe stiffness of 50 pN/µm. The micropipette location was determined using a similar procedure with a three-segment search to find the RBC-micropipette interface (Fig. S6). This appeared brighter in intensity under our microscope on one of these segments (hence, the intensity along the segments did not need to be inverted for maximum location search).

### Microscopy

The experiments were observed with a Nikon TiE inverted microscope (Nikon Instruments, France) using a Nikon 40× 1.3NA S Fluor oil immersion objective enabling UV excitation for calcium imaging. A supplementary 1.5× lens was used, leading to a final magnification of 107 nm/(square) pixel. Alternately a Nikon 60× 1.4NA Plan Apo VC oil immersion objective was used when no calcium measurement were performed. Brightfield, or DIC were used for transmitted light, while calcium ratio imaging was performed with 340 and 380 nm excitation wavelength and 510 nm emission wavelength. A Pinkel fluorescent block (Semrock, US) enabled additional mCherry and FITC channels to be used. A cooled CCD camera (Orca ER, Hamamatsu, France) and a homemade script used with Micro-Manager (http://www.micro-manager.org, [52]) were used for image acquisition. Images were acquired at 0.25 Hz or 1 Hz in absence of Calcium measurement.

### Calcium Imaging

T cells at approximately 10^6^ cells/mL were loaded with 2 µM fura2-AM probe at 37°C for 20 minutes in the dark. Cells were washed and resuspended in MS buffer. A custom-programmed time-lapse used with Micro-Manager enabled the user to define a background region before starting the acquisition. The raw 340 nm- and 380 nm- images were acquired every 4 s using an exposure time of 200 ms. After live background subtraction, the ratio r_fura2_ = 100*F_340 nm_/F_380 nm_ was displayed live using Micro-Manager user interface enabling overlay with brigthfield and other fluorescence channels at will. The multiplication factor 100 appearing in r_Fura2AM_ is arbitrary, though needed for storing a 16-bit numerical value superior to 1. We developed an ImageJ macro to perform post-processing of the ratio images from the raw 340 nm- and 380 nm- images, assuring that the background region of interest was never occupied by a unwanted fluorescent cell.

## Supporting Information

Figure S1 **Anti-CD3 coated beads induce tyrosine phosphorylation of signalling molecules in- and IL2 production by- primary CD4^+^ T cells.** A: CD4+ T cells were incubated with one bead per T cell (anti-CD3 coated or uncoated beads) and lyzed after different times of incubation. Postnuclear lysates were run on SDS-PAGE transferred and blotted with anti-phospho-protein specific antibodies (P-protein) or anti-α-tubulin as a control of charge. B: CD4+ T cells from 3 different donors were incubated with one bead per T cell overnight. IL2 concentration was measured by ELISA in the supernatants of co-cultures.
(TIF)

Figure S2 **Adhesion to silica microbeads does not trigger any [Ca^2+^]_i_ increase.**
(TIF)

Figure S3 **The dynamic-probe protocol does not influence the protrusion average growth speed.** Protrusion length L_protrusion_ and force F_residual_ applied to it during its growth. When “fleeing away” from the growing protrusion, the force probe does not exert forces exceeding typically 25 pN. Furthermore, when transient pulling forces reaching 75 pN are applied the general trend of the growth (i.e. average growth speed) is not perturbed. Inset shows the distribution of the growth speed v_protrusion_ = 0.07+/−0.02 (SD).
(TIF)

Figure S4 **[Ca^2+^]_i_ dynamics are the same upon engagement of CD3 only, or both CD3 and CD18.** Fura2 ratio versus time for T cells contacted by the BFP probe with an anti-CD3+anti-CD18 -coated bead (blue line, error bar is SEM, N = 26) or an anti-CD3-coated bead (black line, error bar is SEM, N = 28). For each curve, the time of the [Ca^2+^]_i_ increase was defined as t = 0.
(TIF)

Figure S5 **Cytoskeleton remodeling observed in fixed T cells.** Beads are coated with either anti-CD3 or anti-CD3+anti-CD18. Polymerized actin (red) and tubulin (green), and brightfield images (right column) are shown. Examples of tube-like (above) or cup-like (middle) protrusions are shown. The third example (below) shows a cell having engulfed a bead.
(TIF)

Figure S6 **Segments used in the tracking procedure superimposed to the bead and probe-holding micropipette.**
(TIF)

Figure S7 **BFP behaves as a linear spring up to a micrometer deformation.** (A) Two-probe procedure consisting in sticking two diametrically opposite RBCs on a single streptavidin-coated bead. One of the probes is stiff (left) relative to the other (right). (B) Deformation of the soft probe (ΔL^soft^_BFP_) versus the deformation of the stiff probe (ΔL^stiff^_BFP_), when one of the RBC-holding pipette is retracted. (C) Predicted and measured value of the ratio k^stiff^/k^soft^ given by Eq. 1 are in excellent agreement (line is of slope 1) (D) Master curve obtained by plotting F^soft^ = k^soft^.ΔL^stiff^_BFP_ versus F^stiff^ = k^stiff^.ΔL^stiff^_BFP_. The straight line has a slope equal to 1.
(TIF)

Movie S1 **T cell interacting with an anti-CD3 coated bead on the force probe.** The probe is not moved once the contact between bead and T cell is achieved. Brightfield images (left) and fura2 ratio (right). Frames are taken every 4 s. Movie is played at 10 frames per second. Bar is 5 µm.
(AVI)

Movie S2 **Large off-axis deviations of the BFP bead during the pushing phase. The bead is coated with anti-CD3. Frames are taken every 4 s. Movie is played at 10 frames per second. Bar is 5 µm.**
(AVI)

Movie S3 **Dynamic-probe protocol used to characterize the pushing phase.** A T cell interacts with an anti-CD3 coated bead on the force probe. Brightfield images (left) and fura2 ratio (right). Frames were acquired every 4 s. Movie is played at 10 frames per second. Bar is 5 µm.
(AVI)

Movie S4 **Dynamic-probe protocol.** Frames were taken every 1 s. Movie is played at 20 frames per second. Bar is 5 µm.
(AVI)

Movie S5 **Pushing phase followed by pulling phase.** Dynamic-probe protocol was used. Brightfield images (left) and fura2 ratio (right). Frames were acquired every 4 s. Movie is played at 10 frames per second. Bar is 5 µm.
(AVI)

Movie S6 **LIFEACT-mCherry T cell interacting with an anti-CD3 coated bead on the BFP.** DIC microscopy (left) and mCherry channel (right). Frames were acquired every 4 s. Movie is played at 10 frames per second. Bar is 5 µm.
(AVI)

Movie S7 **LIFEACT-mCcherry T cell interacting with an anti-CD18 coated bead on the BFP.** DIC microscopy (left) and mCherry channel (right). Frames are taken every 4 s. Movie is played at 10 frames per second. Bar is 5 µm.
(AVI)

Movie S8 **LIFEACT-mCherry T cell interacting with an anti-CD3+anti-CD18 coated bead on the BFP. DIC microscopy (left) and mCherry channel (right). Frames are taken every 4 s. Movie is played at 10 frames per second. Bar is 5 µm.**
(AVI)

Movie S9 **Observation of protrusions with T cells in suspension.** Videomicroscopy experiments were performed using the Leica DM IRBE microscope with the 40× 1.4 NA oil immersion objective. T cells were mixed gently in suspension with anti-CD3 coated beads, put on poly-L-lysine coated coverslips and rapidly placed into a chamber on the microscope at 37°C in a 5% CO2 atmosphere. Phase contrast images were acquired.
(AVI)

Text S1 **Supplementary Materials and Methods.**
(DOC)
