# Supplementary figures and images for: Force Generation upon T Cell Receptor Engagement
Source: PLoS One. 2011 May 10;6(5):e19680. doi: 10.1371/journal.pone.0019680 (PMC3091878; doi:10.1371/journal.pone.0019680)

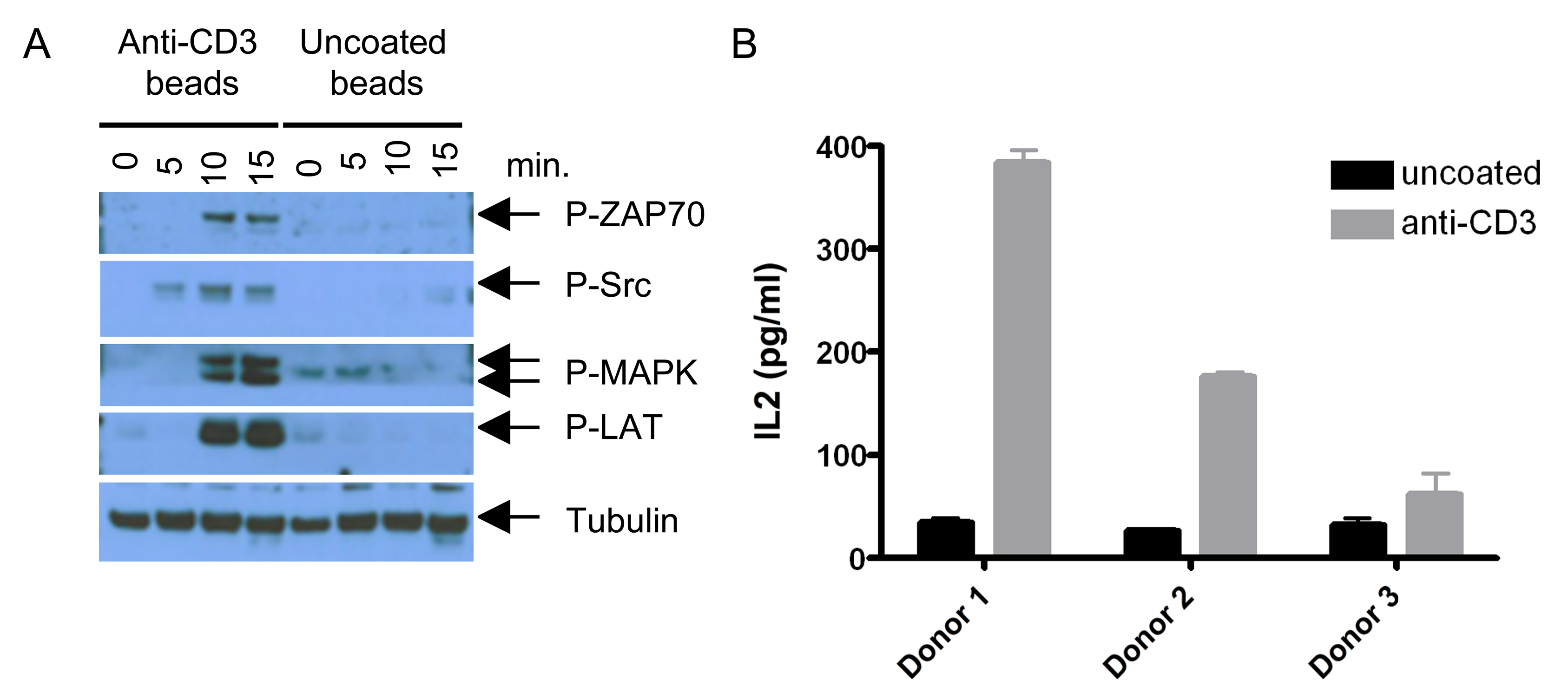

Supplement: Figure S1 — Anti-CD3 coated beads induce tyrosine phosphorylation of signalling molecules in- and IL2 production by- primary CD4+ T cells. A: CD4+ T cells were incubated with one bead per T cell (anti-CD3 coated or uncoated beads) and lyzed after different times of incubation. Postnuclear lysates were run on SDS-PAGE transferred and blotted with anti-phospho-protein specific antibodies (P-protein) or anti-α-tubulin as a control of charge. B: CD4+ T cells from 3 different donors were incubated with one bead per T cell overnight. IL2 concentration was measured by ELISA in the supernatants of co-cultures. (TIF) [file pone.0019680.s001.tif]

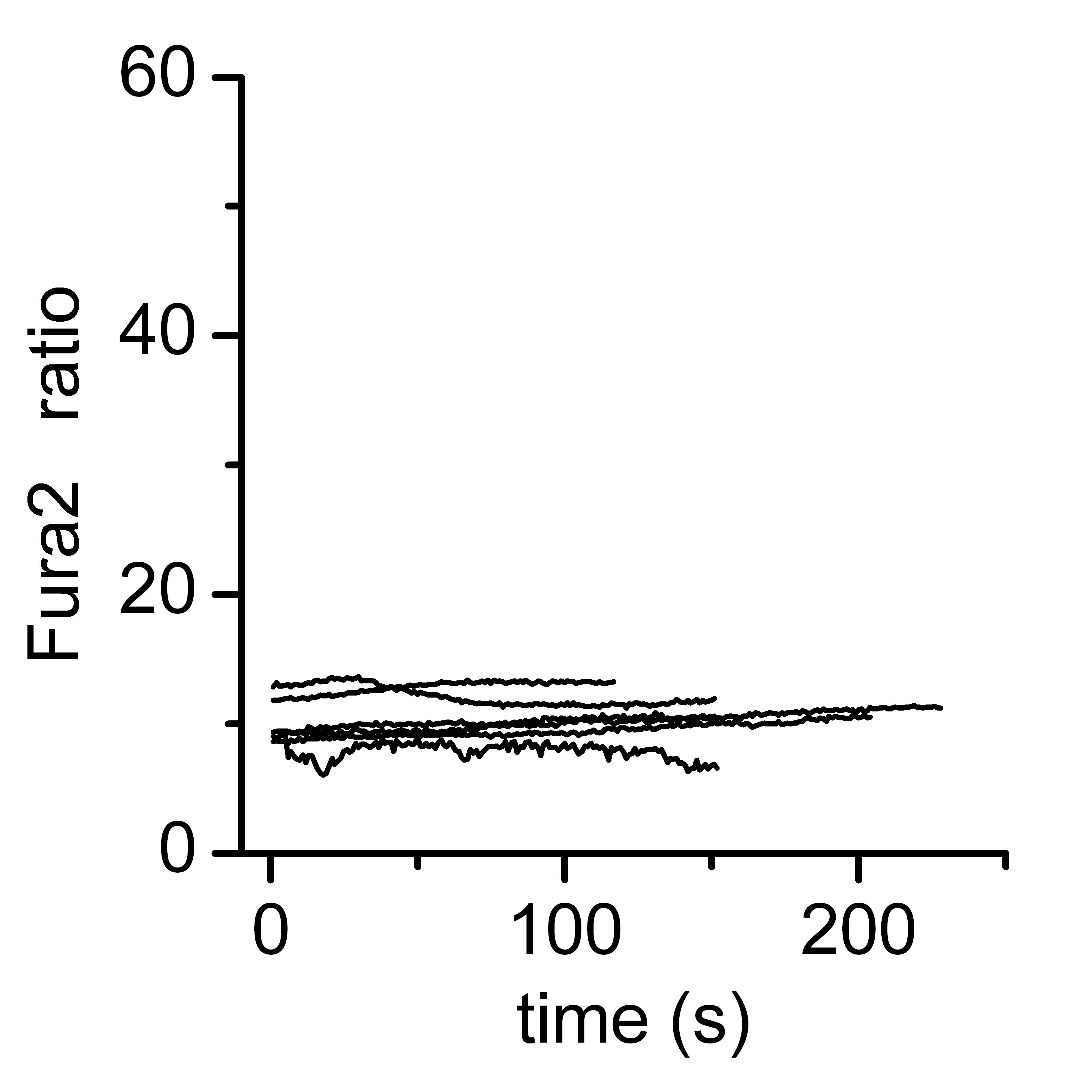

Supplement: Figure S2 — Adhesion to silica microbeads does not trigger any [Ca2+]i increase. (TIF) [file pone.0019680.s002.tif]

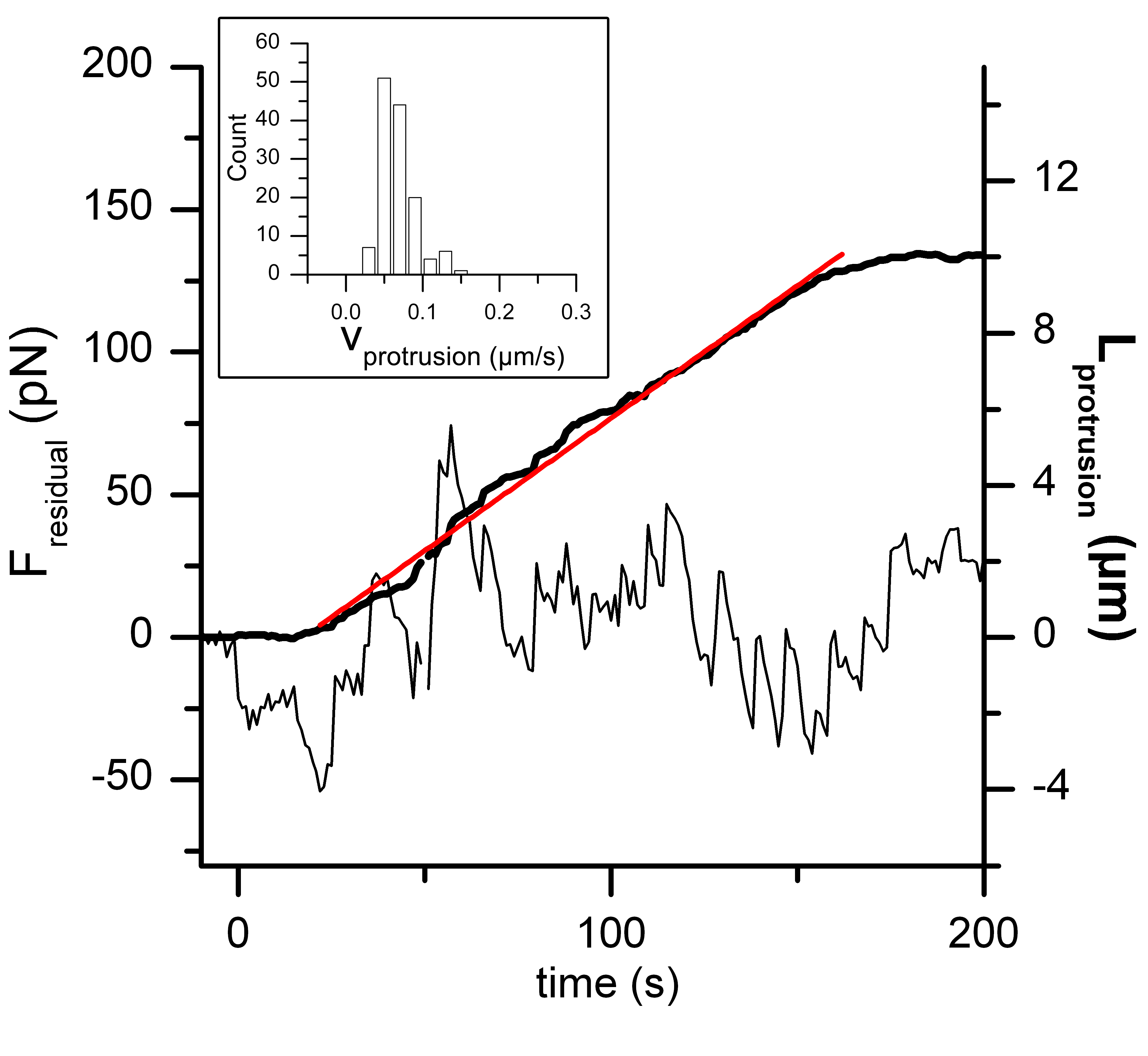

Supplement: Figure S3 — The dynamic-probe protocol does not influence the protrusion average growth speed. Protrusion length Lprotrusion and force Fresidual applied to it during its growth. When “fleeing away” from the growing protrusion, the force probe does not exert forces exceeding typically 25 pN. Furthermore, when transient pulling forces reaching 75 pN are applied the general trend of the growth (i.e. average growth speed) is not perturbed. Inset shows the distribution of the growth speed vprotrusion = 0.07+/−0.02 (SD). (TIF) [file pone.0019680.s003.tif]

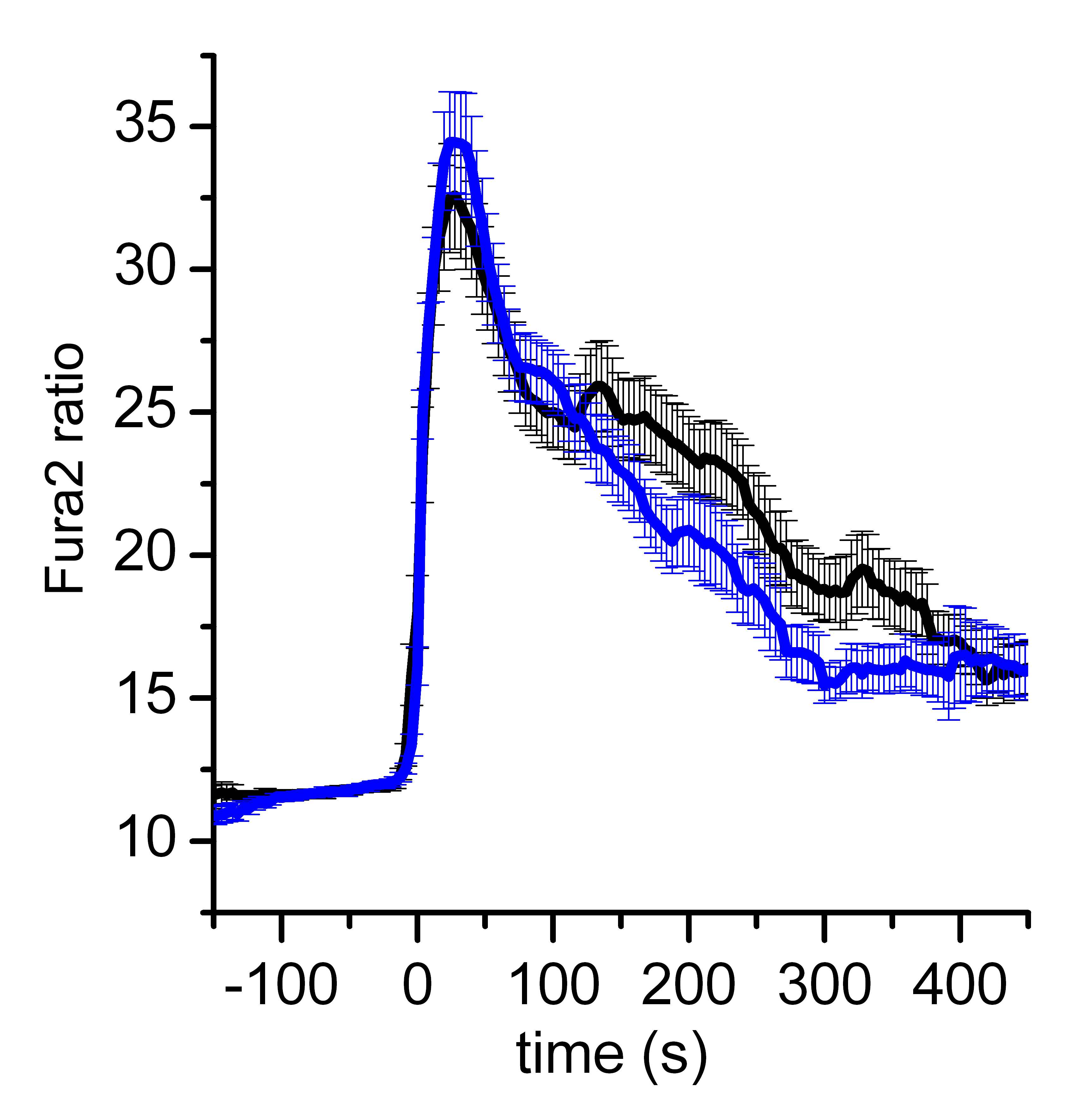

Supplement: Figure S4 — [Ca2+]i dynamics are the same upon engagement of CD3 only, or both CD3 and CD18. Fura2 ratio versus time for T cells contacted by the BFP probe with an anti-CD3+anti-CD18 -coated bead (blue line, error bar is SEM, N = 26) or an anti-CD3-coated bead (black line, error bar is SEM, N = 28). For each curve, the time of the [Ca2+]i increase was defined as t = 0. (TIF) [file pone.0019680.s004.tif]

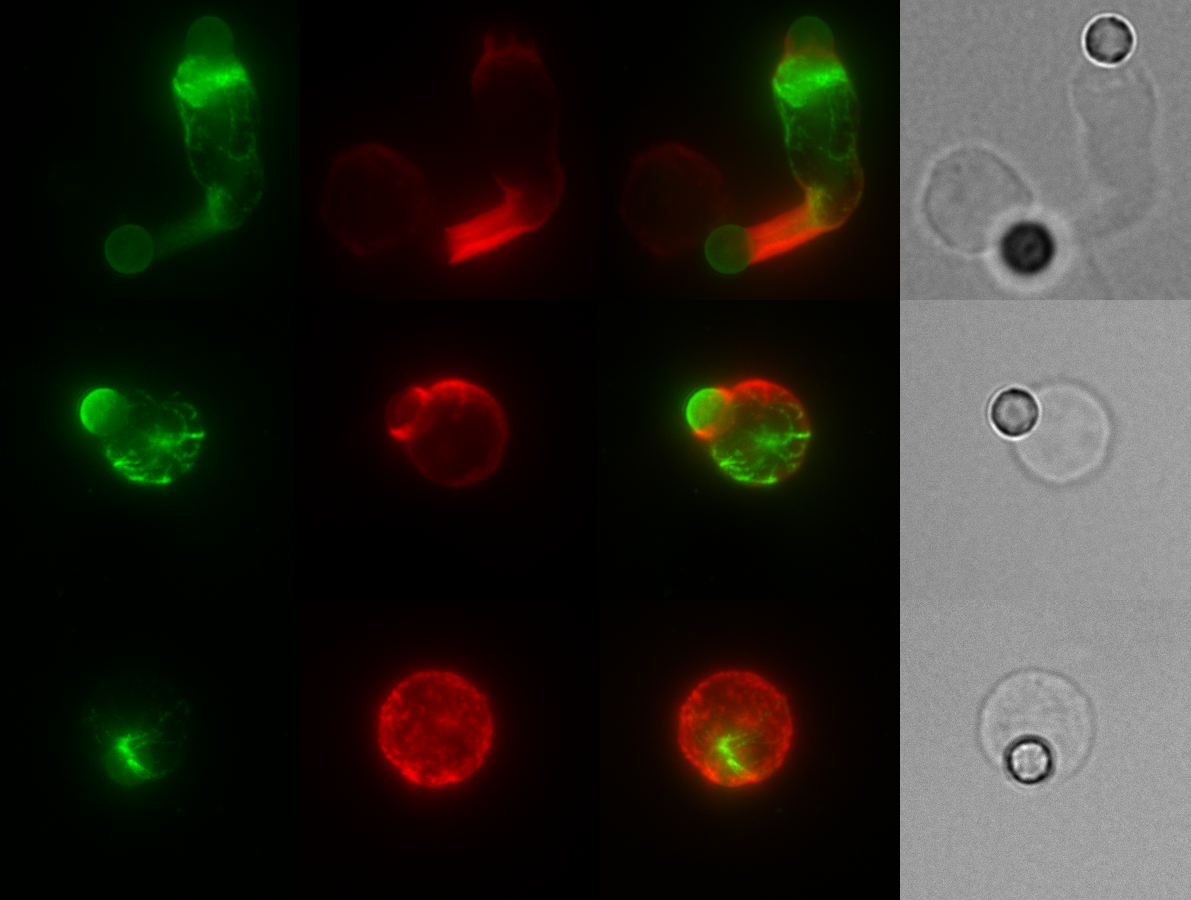

Supplement: Figure S5 — Cytoskeleton remodeling observed in fixed T cells. Beads are coated with either anti-CD3 or anti-CD3+anti-CD18. Polymerized actin (red) and tubulin (green), and brightfield images (right column) are shown. Examples of tube-like (above) or cup-like (middle) protrusions are shown. The third example (below) shows a cell having engulfed a bead. (TIF) [file pone.0019680.s005.tif]

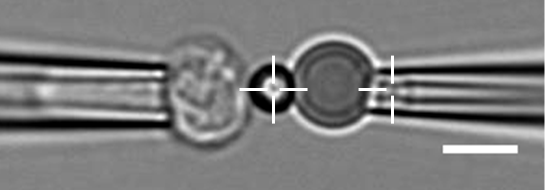

Supplement: Figure S6 — Segments used in the tracking procedure superimposed to the bead and probe-holding micropipette. (TIF) [file pone.0019680.s006.tif]

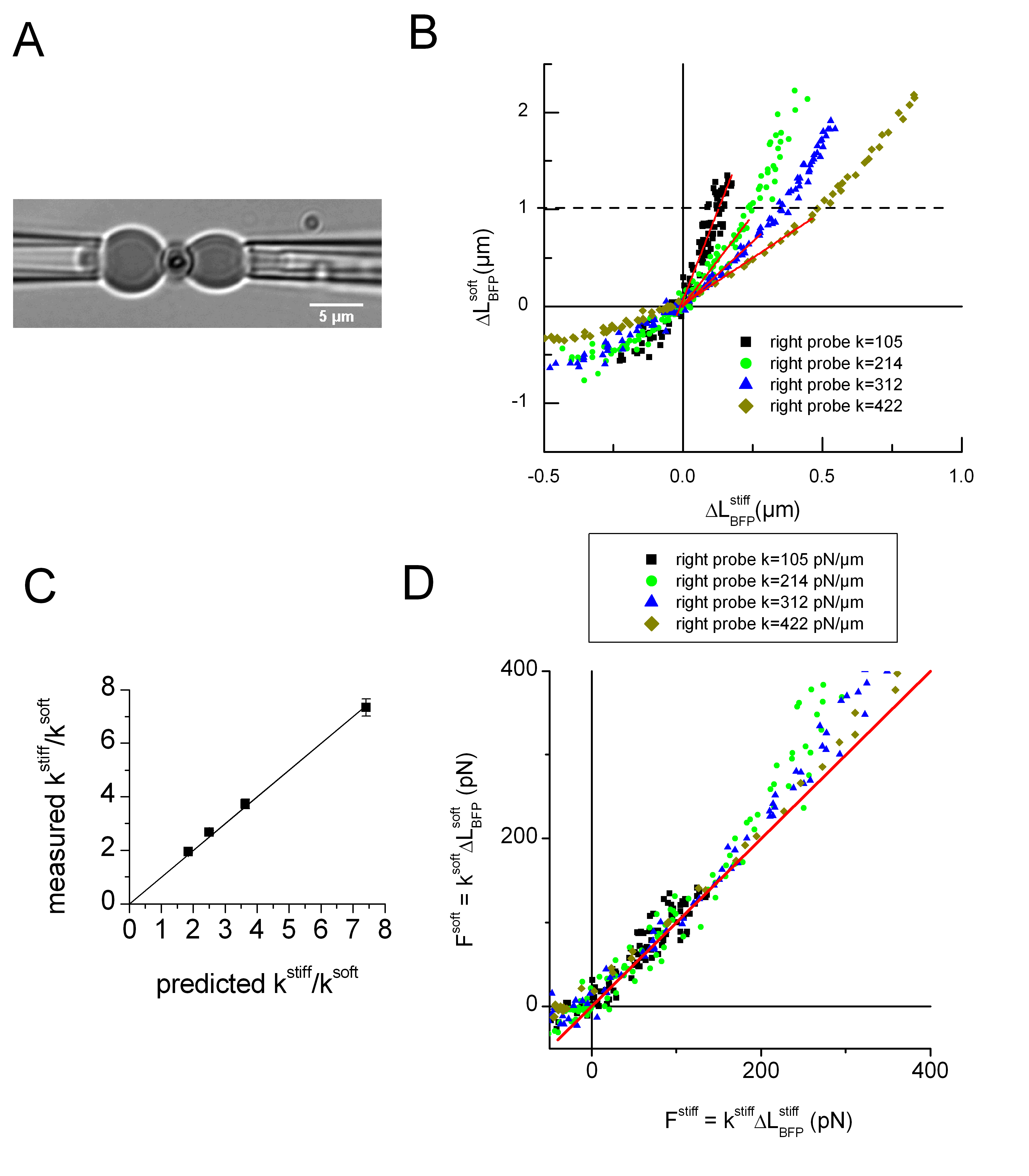

Supplement: Figure S7 — BFP behaves as a linear spring up to a micrometer deformation. (A) Two-probe procedure consisting in sticking two diametrically opposite RBCs on a single streptavidin-coated bead. One of the probes is stiff (left) relative to the other (right). (B) Deformation of the soft probe (ΔLsoft BFP) versus the deformation of the stiff probe (ΔLstiff BFP), when one of the RBC-holding pipette is retracted. (C) Predicted and measured value of the ratio kstiff/ksoft given by Eq. 1 are in excellent agreement (line is of slope 1) (D) Master curve obtained by plotting Fsoft = ksoft.ΔLstiff BFP versus Fstiff = kstiff.ΔLstiff BFP. The straight line has a slope equal to 1. (TIF) [file pone.0019680.s007.tif]
