# Supplementary material for: Force Generation upon T Cell Receptor Engagement
Source: PLoS One. 2011 May 10;6(5):e19680. doi: 10.1371/journal.pone.0019680 (PMC3091878; doi:10.1371/journal.pone.0019680)
Supplement: Text S1 — Supplementary Materials and Methods. (DOC) [file pone.0019680.s017.doc]

# Supporting Text S1. Supplementary Materials and Methods.

Dynamic-probe protocol does not influence protrusion average growth speed.

The dynamic-probe protocol (see text) consisted in progressively stepping back the micropipette holding the BFP as the cell protrusion grew. As soon as RBC compression was detected by the operator, the right pipette was stepped back along the *x*-axis up to the detection of the minimal RBC elongation. We wanted to evaluate the amplitude of the residual forces F_residual_ applied on the probe after its retraction and that they did not influence the average growing speed v_protrusion_ of the protrusion. For this aim, we performed experiments with brightfield image at higher acquisition rate (1 Hz instead of 0.25 Hz, see Movie S4) in order to have a better time resolution. We measured the force F_residual_ exerted at any time by the BFP on the growing protrusion simultaneously with the protrusion length L_protrusion_. Figure S3 shows that during the growth phase (between t=20 s and t=150 s), L_protrusion_ can be considered as linearly increasing with time with a good approximation. The figure shows that the amplitude of F_residual_ is typically of 25 pN. However, at t ≈ 60 s, we deliberately pulled stronger on the protrusion for a short period of time by retracting the pipette further, leading to ≈ 75 pN pulling forces. This higher pulling force did not reflect in a significant change in the average v_protrusion_.

Microscopy on fixed cells

CD4+ T cells in suspension were mixed with coated beads and put on poly(L-lysine)-coated coverslips. After 20 minutes, cells were fixed with 3% formaldehyde (Carlo Erba), incubated in PBS glycine (10 mM) to quench free aldehyde groups, and permeabilized with 0.05% saponin. Cells were then incubated with Alexa546-Phalloidin to label the polymerized actin, and with an anti-α tubulin and an Alexa Fluor 488-labelled secondary Ab, to label the microtubules. Coverslips were mounted onto glass slides using Fluoromount-G (Southern Biotechnology Associates). Images were acquired with a wide-field Eclipse 90i Upright Microscope (Nikon) equipped for image deconvolution. Acquisition was performed using a 100x Plan Apo VC 1.4 Oil objective and a highly sensitive cooled interlined CCD camera (Roper CoolSnap HQ2). Z-positioning was accomplished by a piezo-electric motor (LVDT, Physik Instrument) and a Z-series of images was taken every 0.2 μm. After deconvolution, images were segmented with the multi-dimensional image analysis (MIA) interface running under MetaMorph (Universal Imaging Corporation) based on wavelet decomposition (Racine V, Sachse M, Salamero J, Fraisier V, Trubuil A, Sibarita JB (2007) Visualization and quantification of vesicle trafficking on a three-dimensional cytoskeleton network in living cells. J Microsc 225: 214-28). Images were analyzed with Metamorph and ImageJ software. Pearson's coefficient was quantified using the JACOP plugin from Image J (Bolte S, Cordelieres FP (2006) A guided tour into subcellular colocalization analysis in light microscopy. J Microsc 224: 213-232).

**Videomicroscopy**

For videomicroscopy experiments, we used the Leica DM IRBE microscope with the 40x 1.4 NA oil immersion objective. T cells were mixed gently in suspension with anti-CD3 coated beads, put on poly-L-lysine coated coverslips and rapidly placed into a chamber on the microscope at 37°C in a 5% CO2 atmosphere. Phase contrast images were acquired.

Tracking procedure

When processing a movie, the user of the macro first determines a rough position of the bead outline on key frames. Then for each image, the procedure automatically plots two horizontal (i.e. along x-axis) East and West segments, and two vertical North and South (i.e. along y-axis) segments. The (16-bit) image intensity is then inverted along those four segments, and the intensity profile is fitted to a Gaussian distribution of find the maximum location. West and East maximum location are then averaged to obtain the bead position center location on the x-axis, while the North and South maximum location, once averaged, provide the bead center location on the y-axis. The micropipette location is used by using a three-segment search, starting again from a rough indication by user. Two vertical segments are used to find the micropipettes horizontal inner edges, and a horizontal segment is used to find the RBC-micropipette interface (Fig. S6), which appears as a brighter intensity under our microscope (hence, the intensity over this segments is maximum at the interface and does not need to by inverted for maximum location search).

Linear regime determination: the two-probes procedure

We investigated the range over which the BFP behaved as a linear spring by introducing the following so-called two-probe procedure. We stuck two RBCs diametrically opposite on a single streptavidin-coated bead (Fig. S7A). Each probe was held by a micropipette and aspiration pressures were set to obtain a stiff probe on the left-hand side (k^stiff^=778 pN/µm), and a softer probe (k^soft^ ranging from 105 up to 422 pN/µm) on the right-hand side*.* By retracting one of the pipettes, a pulling force was transmitted to the probes. Both probes, felt the same pulling force F, according to the principle of action and reaction. Hence F = k^stiff^.ΔL^stiff^_BFP_ = k^soft^.ΔL^soft^_BFP_, where k^stiff^ (/k^soft^) is the stiff (/soft) probe stiffness, and ΔL^stiff^_BFP_ (ΔL^soft^_BFP_) is the stiff (/soft) probe elongation. The soft probe extension ΔL^soft^_BFP_=(k^stiff^/k^soft^)ΔL^stiff^_BFP_ varies then linearly with the stiff probe extension, with a constant proportional factor k^stiff^/k^soft^. During the pipette retraction, we measured at each time ΔL^soft^_BFP_ and ΔL^stiff^_BFP_. Plotting ΔL^soft^_BFP_ versus ΔL^stiff^_BFP_ enabled to establish that ΔL^soft^_BFP_ was indeed proportional to ΔL^stiff^_BFP_, for elongations up to ΔL^soft^_BFP_ ≈ 1 µm. Furthermore, the slope was in excellent agreement with the predicted value of k^stiff^/k^soft^ given by Eq. 1 (see Material and Methods), knowing the aspiration pressures and geometrical parameters of both RBCs (Fig. S7C). The soft probe departed from linearity above ΔL^soft^_BFP_ ≈ 1 µm for several values of k^soft^. This 1 µm extension limit leads to a limit in maximal reliably measurable force. This force depends on the used stiffness: it ranges from 50 pN at k = 50 pN/µm to 1000 pN at k = 1000 pN/µm. For probe extensions below this 1 µm threshold, we could collapse several ΔL^soft^_BFP_ vs ΔL^stiff^_BFP_ curves on a F^soft^ vs F^stiff^ master curve, by converting elongations ΔL^stiff^_BFP_ (resp. ΔL^soft^_BFP_) into forces F^stiff^=k^stiff^.ΔL^stiff^_BFP_ (resp. F^soft^=k^soft^.ΔL^stiff^_BFP_). The fact that the slope of this master curve equals unity validates that Fstiff = F^soft^ and is thus consistent with the linear behavior of the RBC as a spring (Fig. S6D).
